# Optimising monitoring efforts for secretive snakes: a comparison of occupancy and N-mixture models for assessment of population status

**DOI:** 10.1038/s41598-017-18343-5

**Published:** 2017-12-22

**Authors:** Robert J. Ward, Richard A. Griffiths, John W. Wilkinson, Nina Cornish

**Affiliations:** 1 Durrell Institute of Conservation and Ecology, School of Anthropology and Conservation, University of Kent, Canterbury, CT2 7NR Kent UK; 2 Amphibian and Reptile Conservation, 655a Christchurch Road, Bournemouth, BH1 4AP Dorset UK; 3 Department of the Environment, States of Jersey, Howard Davis Farm, La Route de la Trinite, Trinity, JE3 5JP Jersey

## Abstract

A fifth of reptiles are Data Deficient; many due to unknown population status. Monitoring snake populations can be demanding due to crypsis and low population densities, with insufficient recaptures for abundance estimation via Capture-Mark-Recapture. Alternatively, binomial N-mixture models enable abundance estimation from count data without individual identification, but have rarely been successfully applied to snake populations. We evaluated the suitability of occupancy and N-mixture methods for monitoring an insular population of grass snakes (*Natrix helvetica*) and considered covariates influencing detection, occupancy and abundance within remaining habitat. Snakes were elusive, with detectability increasing with survey effort (mean: 0.33 ± 0.06 s.e.m.). The probability of a transect being occupied was moderate (mean per kilometre: 0.44 ± 0.19 s.e.m.) and increased with transect length. Abundance estimates indicate a small threatened population associated to our transects (mean: 39, 95% CI: 20–169). Power analysis indicated that the survey effort required to detect occupancy declines would be prohibitive. Occupancy models fitted well, whereas N-mixture models showed poor fit, provided little extra information over occupancy models and were at greater risk of closure violation. Therefore we suggest occupancy models are more appropriate for monitoring snakes and other elusive species, but that population trends may go undetected.

## Introduction

Monitoring populations is crucial for informing conservation measures. The status of a population and the drivers influencing it are often assessed over time using measures of occupancy or abundance^1–3^. These measures vary in terms of the quality and quantity of data needed, and the appropriate monitoring strategy for a population is often unclear^4,5^. The choice of monitoring strategy can result in different status assessments^4^ and must consider that population changes can occur through subpopulation colonisation and extinction, or a decline across the whole population^5^. Occupancy can be assessed as the proportion of an area containing a species, based on repeated observations of presence or absence at a number of sites. Alternatively, abundance measures can make use of counts and vary in their logistical requirements^6^.

Capture-Mark-Recapture (CMR), removal and distance-sampling are common methods for estimating abundance^7,8^, but are time-consuming and not always applicable^9–11^. Indeed for some snake species, recaptures are often low^12,13^. Alternatively, low-cost methods that integrate imperfect detection into presence-absence^14^ and simple count methods (e.g., binomial N-mixture^9^) are attractive and have been shown to provide reliable estimates of occupancy and abundance respectively without the need for individual identification of animals^3,14,15^. Furthermore, as counts are often conducted simultaneously with presence-absence surveys, estimating abundance from counts may require little additional survey effort. With an appropriate level of survey effort, both methods can be used for rare or cryptic populations where detections are low and other, more intensive methods would be unsuitable^6,9,14,16^.

The design of an optimal monitoring scheme can require a choice between measuring occupancy or abundance, and must consider the economic and logistical costs associated with each approach^3–5^. The two measures are closely related but do not provide the same information^16–18^. Moreover, occupancy and abundance can be driven by disparate biotic and abiotic factors, and may therefore be complementary in assessing population status and change whilst informing conservation measures. Occupancy methods may fail to detect changes in population size and therefore underestimate extinction risk if changes in occupancy and abundance are occurring at different rates^19^ (but see ref.^4^). However, they are cost-effective, can aid conservation assessment and be used to monitor cryptic taxa such as snakes^3,16,20,21^. Alternatively, abundance measures can provide information about population size but tend to require greater resources^5,18^, high species detectability^6,22^ and more stringent modelling assumptions^3,9^.

A fifth of reptiles are considered threatened, and a further fifth Data Deficient, due largely to limited data on population trends^19^. Declines have occurred at global^23^ and regional levels^24^, and there is growing concern over potential widespread snake declines^25,26^ with a poor understanding of the underlying causes^8,23,26,27^. Bridging the gap in reptile threat assessment is challenging, with evolutionary and biological traits influencing both extinction risk and our ability to gather appropriate information^28,29^. Attempts to monitor these populations are often carried out by regional or national organisations, such as those in the UK^30^ and the Netherlands^22^. Because of inherent low detectability, reptile surveys often combine visual surveys and the placement of artificial cover objects (ACOs) along transects^31^, which limits the application of traditional distance-sampling approaches.

Snakes have some of the lowest detection rates among reptiles^16^ (perhaps with the exception of fossorial taxa such as Amphisbaenia^19,29^). They can occur at low densities, have wide ranges, cryptic colouration and behaviour, and are often unobservable due to their chosen habitats^16^. They are therefore particularly difficult to study^32^, and previous work has often struggled to attain reliable estimates of snake occupancy, detection and abundance^13,27,33^. This highlights a need to identify the most appropriate tools for monitoring snake populations and optimising detectability^16,19,26^ in order to reduce Data Deficiency in this group.

We test the application of two low-cost approaches to monitoring; occupancy^14^ and binomial N-mixture models^9^ (a count-based method), on two years of survey data of a rare, insular population of grass snakes in Jersey (British Channel Islands)^30,34^. Previous studies of other grass snake populations have found them to be stable^26^ or in decline^35^. However, the species is known to be wide-ranging^36^ and elusive with low to moderate detectability^20,26^, making it difficult to monitor^33^. The status of Jersey’s grass snake population is unknown, and is currently monitored using an occupancy framework under the National Amphibian and Reptile Recording Scheme (NARRS). However, this citizen science scheme recorded only four grass snakes in Jersey between 2007 and 2012^30^ and is likely to have underestimated the species’ distribution. Therefore we assessed the ability of the NARRS protocol to detect grass snakes and changes in its population, and provide recommendations for future monitoring efforts.

## Results

A total of 12,335 ACOs and 613 km of transects were surveyed across the whole study period. We recorded 51 snake observations with an average of one observation every two to three surveys (mean per survey: 0.39 ± 0.07 s.e.m., range: 0–4), and an average of 2.68 (±0.84 s.e.m., range: 0–12) observations per transect in a season. Only 8.3% of survey visits resulted in counts >1. Three observations were sloughed skins and one a carcass. ACOs proved effective in aiding detection, with 76.5% of observations occurring beneath them. A further 15.7% of detections were of basking individuals and 3.9% of active snakes.

### Detection and occupancy

**Figure 1:**
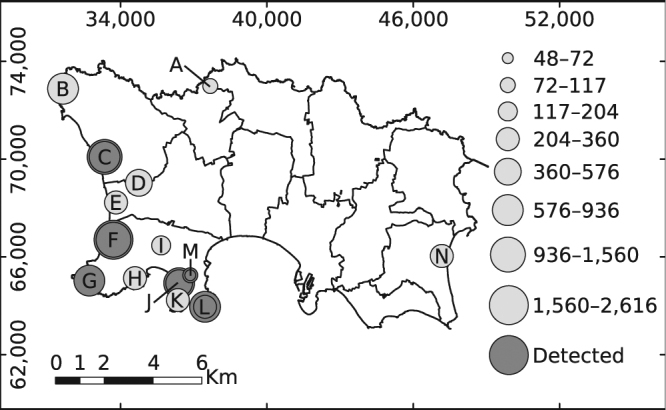
Map of Jersey showing study sites (labelled circles), number of ACOs checked in each season (size of circle) and naïve occupancy (dark grey = occupied, light grey = unoccupied). Sites with concentric circles were surveyed in both years. The map was generated in ArcMap v.10.5 (http://arcgis.com) and refined in Inkscape v. 0.91 (https://inkscape.org).

**Figure 2:**
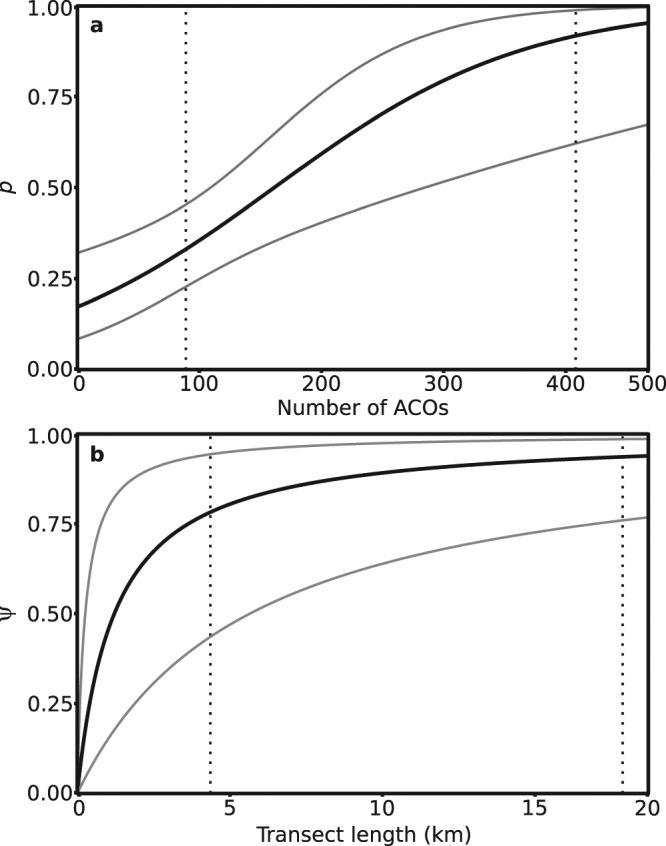
Predicted (**a**) detection and (**b**) occupancy probabilities based on top models (Table 1) with number of ACOs from 0–500 (**a**) or transect length from 0–20 km (**b**). Models shown are (**a**) *p*(ACOs), ψ(habitat) and (**b**) *p*(ACOs), ψ(.). Grey lines indicate 95% confidence intervals. Vertical dotted lines show mean (left) and maximum (right) (**a**) number of ACOs or (**b**) transect lengths used in this study. The figure was generated in R (R Core Team (2015). R: A language and environment for statistical computing. R Foundation for Statistical Computing, Vienna, Austria. URL: https://www.R-project.org/) using package ggplot2 v. 2.2.1^68^ and refined in Inkscape v. 0.91 (https://inkscape.org).

Snakes were detected at 11 out of 19 study sites, with no changes in observed occupancy between years (Fig. 1). Goodness-of-fit tests indicated good model fit, and ranking identified one top detection and one top occupancy model with ΔAICc < 2 (Table 1). Mean detectability (i.e., the probability of detecting at least one grass snake along a transect during a single survey if they were present, *p*) was estimated at 0.33 (±0.06 s.e.m.) which was greater than our observed detection rate of 0.25 (±0.04 s.e.m) across all surveys (including from potentially unoccupied sites). The mean estimate of occupancy (ψ) per km of transect was 0.44 (±0.19 s.e.m.). However, the probability of a transect being occupied increased with its length (Fig. 2) and the mean probability of any site’s total transect length being occupied was 0.67 (±0.05 s.e.m.; Table 2), which was higher than our naïve transect occupancy of 0.58 (Fig. 1).

**Table 1:** Top models (ΔAICc < 2) of detection (*p*), occupancy per km (ψ) and abundance per km (λ) for grass snakes in Jersey. Parameters (*p*, ψ and λ) were constant (.) or allowed to vary with covariates. Due to the small sample size, models are ranked by their AICc and weight (w_*i*_). Models are shown for number of observations set to 132 (total number of surveys) for detection and 19 (number of sites) for occupancy and abundance. All models include an offset for transect length on occupancy or abundance. *N* is number of parameters in the model and LL the log-likelihood. The mean prediction and its standard error are shown for each parameter. Goodness-of-fit statistics are also shown.

Model	*N*	AICc*	ΔAICc*	w_i_*	LL	Prediction	Goodness-of-fit		
							χ^2^	*p*	$\hat{c}$
**detection**									
*p*(ACOs), ψ(habitat)	6	124.31	0.00	0.55	−55.82	0.33 (0.06)	291.64	0.80	0.84
**occupancy (per km)**									
*p*(ACOs), ψ(.)	3	127.88	0.00	0.65	−60.14	0.44 (0.19)	325.41	0.63	0.91
**abundance (per km)**									
*p*(.), λ(.)	3	166.11	0.00	0.42	−79.26	0.44 (0.14)	250.09	0.01	1.96

*Due to overdispersion, the abundance model rankings are instead QAICc, ΔQAICc and QAICc weight.

**Table 2:** Study sites in Jersey, Channel Islands sampled in 2014 and 2015 showing: year sampled, dominant habitat type, site area in hectares (ha), number of surveys (*K*), number of ACOs and length of transect surveyed in kilometres (km). Transect-specific estimates of detection (*p*), occupancy (ψ), and abundance (λ) are shown with 95% confidence intervals.

Site	Year	Habitat^a^	Area (ha)	*K*	ACOs	Transect length (km)	*p*	ψ	λ
A	2015	SC	8.10	3	39	2.91	0.23 (0.13‒0.36)	0.69 (0.33‒0.91)	0.67 (0‒3)
B	2014	SC	75.91	8	90	6.07	0.32 (0.22‒0.44)	0.82 (0.50‒0.96)	0.47 (0‒2)
C	2014	DGr	36.99	8	117	6.40	0.38 (0.27‒0.50)	0.83 (0.51‒0.96)	2.94 (2‒5)
C	2015	DGr	36.50	6	238	10.53	0.64 (0.43‒0.81)	0.89 (0.64‒0.97)	7.94 (5‒11)
D	2014	SC	23.19	8	72	3.41	0.29 (0.19‒0.41)	0.72 (0.36‒0.92)	0.27 (0‒2)
E	2014	AGr	29.63	8	34	1.12	0.22 (0.13‒0.36)	0.46 (0.16‒0.80)	0.09 (0‒1)
F	2014	DGr	65.72	8	195	8.77	0.55 (0.38‒0.71)	0.87 (0.59‒0.97)	5.44 (4‒8)
F	2015	DGr	65.64	6	436	19.10	0.92 (0.62‒0.99)	0.94 (0.76‒0.99)	8.06 (5‒12)
G	2014	SC	38.81	8	81	4.19	0.30 (0.20‒0.43)	0.76 (0.41‒0.94)	1.33 (1‒3)
H	2014	RGr	29.53	8	45	4.51	0.24 (0.14‒0.37)	0.78 (0.43‒0.94)	0.35 (0‒2)
I	2015	AGr	3.89	4	51	1.76	0.25 (0.15‒0.38)	0.58 (0.23‒0.86)	0.33 (0‒2)
J	2014	SC	10.48	8	60	2.20	0.26 (0.17‒0.39)	0.63 (0.27‒0.89)	1.54 (1‒3)
J	2015	SC	10.48	6	110	4.86	0.36 (0.25‒0.48)	0.79 (0.45‒0.95)	4.14 (2‒7)
K	2014	SC	15.77	8	42	2.80	0.23 (0.14‒0.37)	0.68 (0.32‒0.91)	0.22 (0‒1)
L	2014	SC	37.54	8	90	6.07	0.32 (0.22‒0.44)	0.82 (0.5‒0.96)	1.79 (1‒4)
L	2015	SC	3.61	6	46	1.46	0.24 (0.14‒0.37)	0.53 (0.19‒0.84)	1.18 (1‒2)
M	2014	RGr	1.01	8	6	0.28	0.18 (0.09‒0.33)	0.18 (0.04‒0.50)	1.08 (1‒2)
M	2015	RGr	1.01	5	18	0.44	0.20 (0.10‒0.34)	0.25 (0.07‒0.61)	1.07 (1‒2)
N	2014	AGr	36.06	8	36	1.62	0.22 (0.13‒0.36)	0.56 (0.21‒0.85)	0.13 (0‒1)

^a^Habitat classifications; AGr = Amenity grassland, DGr = Dune grassland, RGr = Rough grassland, SC = Scrub.

Occupancy and detection estimates were variable between sites. For sites surveyed in both years, estimates of occupancy were fairly stable between years whereas detection varied with survey effort (Table 2). However, site L had a much lower occupancy probability in 2015 due to a 75.9% reduction in transect length. Detection increased with survey effort (number of ACOs) (Fig. 2), whereas occupancy showed no influence of covariates (Table 1). We failed to identify any environmental covariates that reliably described detection.

### Abundance

All model mixtures gave similar predictions, AICc rankings and showed evidence of poor performance and overdispersion (see Supplementary Table S1). However, we continue with the use of the Poisson distribution for estimating abundance (λ) as the negative binomial mixture can often be unreliable^17,37^. A single model giving constant detection and abundance had ΔQAICc ≤ 2 (Table 1).

Transect-specific empirical Bayes estimates of abundance were typically low (mean: 2.05 ± 0.58 s.e.m., range: 0.1–8.1; Table 2). Collectively they provided a total estimated abundance of 39 (95% CI: 20–169) snakes associated with the study transects. For sites surveyed in both years, estimated abundance was consistent for sites L and M, but varied between years due to changes in transect length for other sites (Table 2). During the study, 43 unique individual snakes were identified based on ventral patterns. Therefore we are able to raise our lower confidence bound of total abundance across transects to 43 individuals.

### Survey effort requirements and recommendations

**Figure 3:**
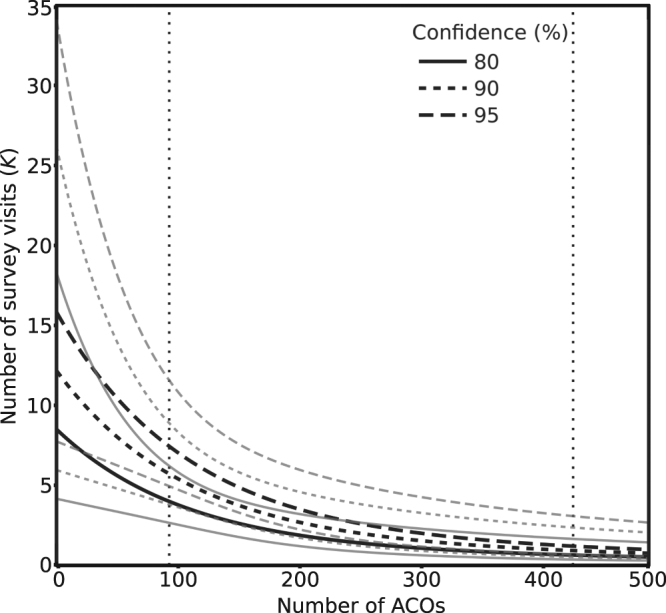
Number of survey visits (*K*) required to determine species presence along a transect with a given probability with number of ACOs from 0–500. Grey lines show 95% confidence. Vertical dotted lines show mean (left) and maximum (right) number of ACOs used in this study. The figure was generated in R (R Core Team (2015). R: A language and environment for statistical computing. R Foundation for Statistical Computing, Vienna, Austria. URL: https://www.R-project.org/) using package ggplot2 v. 2.2.1^68^ and refined in Inkscape v. 0.91 (https://inkscape.org).

The number of survey visits required to have confidence in species absence is highly dependent upon survey effort and associated species detectability (Figs 2‒3). With our mean survey effort of 95 ACOs per site and a predicted detection (*p*) of 0.33 (95% CI: 0.23–0.45), four (95% CI: 3–6), six (95% CI: 4–9) or seven (95% CI: 5–12) site surveys would be needed for 80, 90 or 95% confidence of absence respectively. In comparison, the current NARRS survey effort of 10 ACOs per site gives a detection estimate of *p* = 0.19 (95% CI: 0.10–0.33) and would require eight (95% CI: 4–16), 11 (95% CI: 6–23) or 15 (95% CI: 7–30) surveys for the same three levels of confidence of absence. For the surveys conducted in this study, at the 80% confidence level, four of the 19 sites were not surveyed sufficiently to declare absence with confidence (Table 2). This increased to nine sites requiring further surveys for 90% confidence and 13 sites for 95% confidence. Snakes were not detected at six of these 13 sites, so we should not declare them absent without further survey effort.

**Figure 4:**
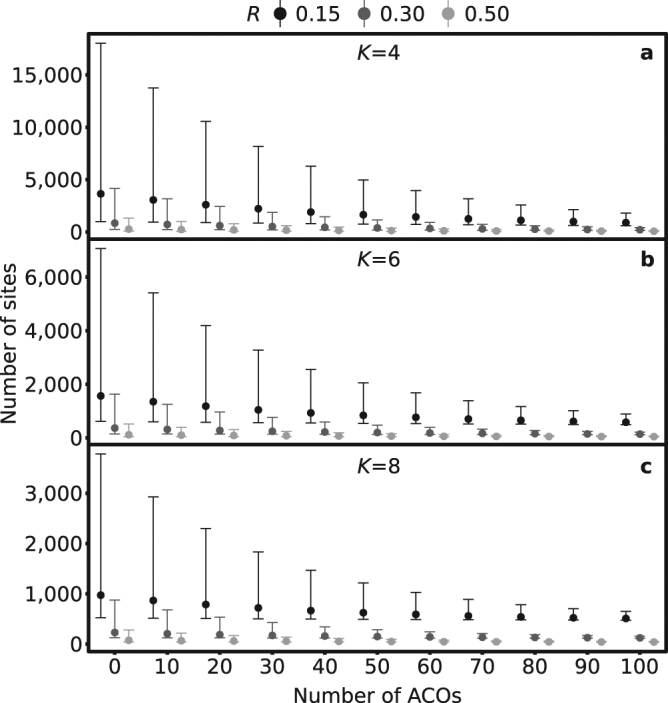
Number of survey sites required to detect a decline in occupancy (ψ) at different levels of survey effort (numbers of ACOs) with varying proportional changes (*R*) in occupancy at a power of 0.8, and (**a**) four, (**b**) six or (**c**) eight survey visits (*K*). Figure displays number of sites required when alpha is set to 0.05 with bars showing 95% confidence intervals. The figure was generated in R (R Core Team (2015). R: A language and environment for statistical computing. R Foundation for Statistical Computing, Vienna, Austria. URL: https://www.R-project.org/) using package ggplot2 v. 2.2.1^68^ and refined in Inkscape v. 0.91 (https://inkscape.org).

For NARRS to be able to detect an occupancy decline with 80% power, we found the number of survey sites needed to be prohibitively large given the current sampling of ≤50 sites per six-year cycle. For example, with the current Jersey NARRS effort of ca. 10 ACOs per site and four surveys, 709 sites (95% CI: 222–3166) are needed within a six-year cycle to detect a 30% decline. By increasing the number of sites surveyed within each survey cycle, the number of ACOs at each survey site or the number of times a site is surveyed, the ability to detect smaller declines improves. To detect any decline with four surveys at ≤50 sites per sampling period (mean number of sites needed: 76, 95% CI: 49–160) and 80% power, at least 90 ACOs are needed per site, and even then a 50% decline may go undetected. A more achievable level of survey effort would be a design where 57 (95% CI: 41–138) sites are surveyed within each cycle with 30 ACOs and eight repeat visits; but this would still only permit a 50% decline to be detected (Fig. 4; Supplementary Table S2).

## Discussion

We applied two commonly used methods for assessing population status in long-term monitoring programmes to a population of cryptic and elusive snakes and estimated their site occupancy, detectability and local abundance. Our findings suggest that both occupancy and N-mixture models can be used to assess the current status of populations with small sample sizes. However, statistical power to detect occupancy declines will be poor^38^ and parameter estimates may exhibit wide confidence limits. Unlike some previous studies, we did not encounter problems with N-mixture models yielding confidence limits including zero^39^ or convergence issues^6^. Nevertheless, although occupancy models showed good model fit to the data, the fit of N-mixture models were less satisfactory and were less informative (Table 1).

We selected semi-natural sites and transects within them *a priori* which were likely to have higher rates of occupancy, abundance and therefore detectability than the wider island landscape. This allows our results to be more effective in informing management actions than if sites had been more widely distributed^3^, but limits our ability to generalise any findings across Jersey due to differences in landscape and survey effort. As we focused on the ‘best’ sites in the island, we infer that grass snake occupancy and abundance elsewhere in Jersey will be lower than our estimates.

Our estimates of detection were lower than a previous grass snake study that used less ACOs^20^, but higher than those from Kéry^40^ that only used visual searches. The top model suggests that detection primarily increases with survey effort^20,41,42^, despite environmental, demographic and physical factors also expected to have an influence^40,41,43–46^. We did not test for demographic effects due to sample size restrictions, and other influences may have gone undetected due to low abundance and a limited population available for detection^47,48^. Previous studies of low-density snake populations may have struggled with N-mixture models due to lower individual detection rates than we experienced, driven in part by less-intensive monitoring efforts and high mobility of study species^33,39^. In these cases detection may be improved by greater survey intensity, using radio-telemetry to identify optimal placement of ACOs or trap arrays, or through novel means such as detector dogs^49^.

Detection plays a key role in determining the presence or absence of a species at a site. In Britain, guidelines recommend four to five site surveys with 30 ACOs to achieve a 95% confidence of grass snake absence^20^, increasing to seven or more at marginal sites^31^. We observed similar requirements for site visit numbers, but only if we consider our sites to be marginal and use larger quantities of ACOs. Furthermore, our results indicated that the current NARRS effort of four site visits per season would be insufficient for assuming grass snake absence from a site with any reasonable confidence. Future analysis of NARRS occupancy data should therefore account for imperfect detection to resolve these issues, or the number of surveys at a site should be increased appropriately to limit the possibility of non-detection. With so little semi-natural habitat remaining, it is vital that sufficient effort is used before sites are designated as absent; particularly where development may occur.

In our study we found a greater area occupied than expected based on results from previous monitoring efforts^30^. This was driven by our improved ability to detect the species due to an intensive survey effort. Simulations have shown occupancy estimates to be fairly unbiased when *p* > 0.3 and there are five or more surveys^14^, therefore our occupancy estimates are likely to be unbiased. However, populations with extremely low detection may result in biased occupancy estimates^14^.

The efficiency of presence-absence over count-based methods can be improved by using a ‘removal’ design^3^, whereby some or all sites are surveyed until a single detection occurs or a given number of surveys are completed. This method would have allowed us to reduce our survey effort from 132 surveys and 12,335 ACO checks, to 90 surveys and 6,556 ACO checks. However, this would lower detection estimates, increase occupancy estimates and generally create greater uncertainty. It may also be unsuitable for multi-species monitoring.

A primary aim of monitoring is to detect population changes and subsequently inform management. Our study indicated that there is very poor power to detect occupancy declines, even when improvements are made by increasing the number of survey sites or ACOs. However, increasing the number of surveys carried out at each site may give a more practical and cost-effective solution to detecting declines (see Supplementary Table S2). Moreover, as our estimates of detection and occupancy were based on surveys carried out in suitable habitat, monitoring carried out over a larger, less suitable landscape with reduced occupancy would likely reduce power further. Consequently, for rare species or those with small population changes, it may not be possible to have sufficient power to detect trends^5,6^.

This is the first study to estimate abundance of grass snakes with N-mixture models, although others have been successful in using CMR^12^. Despite not encompassing the whole island, our estimates suggest a very small population inhabits the remaining suitable habitats. Previous simulation studies found there to be only a small positive bias in mean abundance with similar sample sizes^9^. However, wide confidence limits in this, and previous studies^39,50^ will afford little power to detect trends. This uncertainty in our abundance estimates may arise from several factors. These include unmodelled heterogeneity in detection between individuals, risk of temporary emigration (e.g., use of burrows), non-independence of sites sampled in both years and only a single recapture amongst the 43 identified snakes occurring across the whole survey effort. Very low individual detection and recapture rates means CMR may be unsuitable for populations of elusive snakes. Considering these issues, it is likely that our abundance estimates are negatively biased, and may be further confounded by having an unknown effective sampling area associated to a transect unless a site is saturated with survey effort^17,22^. This issue can only be remedied through the use of logistically demanding spatial capture-recapture or distance-sampling approaches^17^. Nevertheless, as a range-restricted insular population it is probable that a regional classification of Vulnerable according to IUCN category D applies^1^ and that there is risk of the population becoming non-viable and extirpated from sites due to low abundance^51^.

Variability in the size of study sites has several implications for conservation when assuming a site is or is not occupied by a species of conservation interest. Small sites may hold fewer individuals which may be more easily extirpated by stochastic events, may have resources that are only used seasonally, have little contribution to the overall population and may only be occupied by an unsustainable sink population. Larger sites may therefore be given greater priority for protection, management and monitoring as they will often contain a wider selection of resources and buffer stochastic losses. However, small populations, particularly those occurring within fragmented landscapes, may depend on a collection of small sites rather than single large ones in order to meet resource requirements and disperse through the landscape. Their importance to conservation objectives therefore should not be solely assessed based on occupancy status at a single point in time, but instead over time with additional complementary information such as resource use and seasonal movement. The way in which the cumulative collection of sites supports the overall population in the long-term should be the main focus.

To avoid bias in population estimates from all model types, the assumptions of those models must be met. The biological reality is that without controlled experiments, assumption violations are likely to occur. Mobile study organisms such as snakes provide several challenges for monitoring studies^39^, including the potential to violate closure assumptions by leaving sites or concealing themselves^47^. These movements can be considered as temporary emigration which can be driven by variation in environment, season or lifestage^6^. Furthermore, on publicly accessible sites such as those studied here, seemingly unobtrusive recreational activities can disturb snakes^52^. Studies on these effects are lacking, however it is likely that they could influence detectability, emigration and survival. For example, massasauga rattlesnakes (*Sistrurus c. catenatus*) move away or conceal themselves more when disturbed by humans^53^.

To meet site closure and independence assumptions^9,47^, sites should be an appropriate size^22,54^, a robust survey design should be used where repeat surveys are carried out in a short time-frame^13,17,55^, and sites should be sufficiently separated to prevent movement between them within a season^3,14,33^. Within Europe, herpetofauna monitoring schemes rarely use the robust design^22,31^, risking closure violation. However, if surveys are conducted in close succession then independence between them may be lost and seasonal effects upon detection may not be evaluated. Moreover, logistical constraints may limit the ability of surveyors to visit a site regularly. Datasets may therefore require truncation or pooling to meet closure assumptions^3,22^. A parallel study (Ward *et al*., in prep.) carried out short-term radio-tracking of 16 adult grass snakes at three study sites in Jersey. The results indicate that the snakes exhibit site fidelity and small ranges, but within the site may be undetectable as they undergo a form of temporary emigration in which they are concealed within burrows or dense vegetation 84% of the time. Where datasets are sufficient, models that account for birth, death and temporary emigration may improve abundance estimation for rare and elusive species^47,54,56^.

Few studies have compared occupancy and N-mixture models (but see ref.^50^), instead focusing on differences between abundance methods^6,41,56^. Due to their comparatively low cost however, presence-absence and count based methods may be the most appropriate for monitoring multiple species, or those with difficult traits such as low detection and high mobility. Of these low-cost options, this study indicates that occupancy frameworks are more appropriate than count based methods for snakes and other elusive and mobile species^13,16,57^ due to (i) low frequency of encounters; (ii) low site abundance with little variation in counts^5,56^; (iii) unresolved issues in N-mixture modelling such as choice, fit and convergence of different error distributions^37,42^; (iv) ability to meet model assumptions; and (v) resource requirements^5,18,57^. Consequently, although they required little extra survey effort, including the count data in this study contributed limited additional and potentially unreliable information on population status.

Monitoring comes in many forms, and may be carried out by academics, citizen scientists, non-governmental organisations, consultant ecologists or other interested parties. Generally all efforts will be to determine population status, but at different scales and with different resources and intensities. Therefore broader recommendations can be gleaned from other simulated and real-world studies. These indicate that occupancy measures tend to be more suited to widespread monitoring and rare species than abundance measures. This is particularly true when there is low cost associated with sampling, a small monitoring budget, many sites, a high frequency of sampling, few observations with little variation and low detection probability^4,5,57,58^. Depending on budget, occupancy measures may detect changes in population size or area occupied respectively^4,18^, and may be best suited to detecting unrelated losses of subpopulations rather than a single synchronous decline^5^. However, the efficiency of occupancy measures may decrease with increasing scale as more effort is taken to visit each site^57^ unless the removal design is used^3^. Furthermore, these measures may have limited power to detect changes, and exhibit sensitivity to the number of observations per site and sampling frequency^5,57^. Abundance measures are better suited when there are fewer sites (≤150), high detectability, high costs associated with sampling, and observations are variable but well explained by covariates^4,5,22,57^. Identifying the thresholds at which these methods are no longer feasible for snakes would be a useful step^33^, as done for birds, which showed abundance surveys to be more cost-effective than occupancy when the species was detected at >16 sites in a season^4^.

In order to make use of scarce resources, monitoring programmes are often designed for multiple widespread species instead of species-specific programmes of umbrella or indicator species^58^. Generally speaking, the former approach whilst utilising citizen scientists may have greater benefits for biodiversity^58^ and is the most cost-effective^59^. Attempts to monitor rare and range-restricted populations may require different approaches due to their spatial scale, number of occupied sites and as in this study, a large investment at each visit in order to get observations^4^. As an example, the Jersey NARRS scheme is aimed at multiple widespread species and benefits from low costs for visiting sites due to the island’s small spatial scale. With sufficient resources, many sites could be sampled making occupancy the appropriate choice. However, as a small island, there are a limited number of sites, volunteers and resources. Even if all available 1 km^2^ cells were surveyed (n = 140)^30^ each year (an unlikely feat), NARRS could still only survey a maximum of 840 sites in a six-year cycle, many ACOs would be needed, and few sites are likely to be occupied by grass snakes. This may confound attempts to make reliable assessments of population status and change^30^ (see Supplementary Table S2). Conversely, with such a limited area it may be possible to survey a larger and more representative proportion of the overall landscape than on the mainland. Applying these issues to other hypothetical restricted populations where the costs of monitoring are greater, or fewer resources are available, the most appropriate monitoring strategy may differ. Indeed, where a high intensity of sampling is required for monitoring as with many snakes^41^ and other elusive species, widespread citizen science programmes may not be suitable. Therefore without specific investment, and noting a lack of power, detecting trends in Jersey’s grass snake population is unlikely. To improve the ability of NARRS to monitor Jersey’s grass snake population, we recommend that efforts are made to enhance species detection at each survey site through increases in the number of ACOs and the number of survey visits. A robust sampling design^55^ will aid statistical analyses, and these methods will generally provide reliable results for other reptile species^20^. Incorporation of a partial or full removal design^3^ may also be beneficial if the primary aim is to ascertain occupancy status.

In summary, few long-term studies of snake populations have been conducted, with available examples using simple counts or CMR to estimate abundance^26,41^. We recommend the incorporation of detection and the influences of covariates upon simple count data to provide more reliable population assessments whilst monitoring at larger scales than possible by CMR and other high-intensity methods. At larger scales still, where closure violation is a risk, or monitoring costs are reduced by using a removal design, occupancy provides a suitable method. Further work comparing the accuracy of different parameter estimates would be useful and could be carried out through simulation^6,57^.

For our study population, the remaining semi-natural areas containing structurally diverse habitats in the west and south-west of Jersey have maintained their occupancy status from previous unpublished work in 2002 (States of Jersey Department of the Environment, pers. comm.). This highlights the importance of this region for the locally scarce grass snake population and warrants further study to inform conservation management of these sites. We encourage others to carry out pilot studies and power analysis during development of monitoring schemes, and to test the application of N-mixture models on small populations where conventional CMR methods are unsuitable. Generally however we recommend the use of occupancy methods for rare and elusive species. Providing data that can enable reliable assessment of snake populations should be prioritised to assess status and investigate potential declines.

## Materials and Methods

To identify the best strategy for monitoring Jersey’s grass snake population and determine current population status, we intensively surveyed remaining habitat over two years. We evaluated the goodness-of-fit and applicability of occupancy and N-mixture models to our data, and identified the factors influencing species detectability. This enabled calculation of the survey effort required to determine absence from a site, and the number of sites to be surveyed to detect an occupancy decline.

### Surveys

The island of Jersey (49°12′N, 2°8′W) is 117 km^2^ and lies 22 km west of Normandy, France. The main pressures to its biodiversity are anthropogenic, with 83% of land-cover modified for human use^60^. We selected 14 study sites (Fig. 1) based upon grass snake distribution data from previous monitoring^30^, the local biological records centre (http://jerseybiodiversitycentre.org.je/) and expert opinion. These were largely within remaining semi-natural areas of Jersey National Park in the west of the island where the species has historically persisted^34^. Sites comprised a mixture of dune grassland, coastal plains, heath and scrub along with amenity grassland and semi-urban areas, and were assigned to one of four habitat classes; amenity grassland, dune grassland, rough grassland or scrub (Table 3). Sites were deliberately large to meet closure assumptions (mean: 27.9 ha, range: 1.01–75.91; Table 2), were delineated by (i) boundaries in land management, (ii) changes in vegetation composition and/or (iii) the presence of barriers to movement (e.g., roads), and were considered to contain all necessary resources for a population.

**Table 3:** Covariates evaluated as potential predictors of grass snake occupancy (ψ), detection (*p*) or abundance (λ). The level indicates whether the covariate was measured at the site-level (site), or within each survey visit (survey). Continuous covariates were scaled to their mean and one standard deviation.

Covariate	Type	Level	Description
habitat^a^	Factor	Site	Habitat type categorised by dominant habitat class calculated in ArcMap 10.2.1 from Phase 1 survey data provided by the Jersey States Department of the Environment: ‘Amenity grassland’, ‘Dune grassland’, ‘Rough grassland’, ‘Scrub’
ACOs	Continuous	Site	Number of artificial cover objects (ACOs) surveyed on each site visit
aspect^a^	Factor	Site	Mean aspect azimuth of site calculated using script from Carl Beyerhelm, Coconino National Forest [Available from: https://geonet.esri.com/thread/47864] (accessed 31/12/2015) in ArcMap 10.2.1: ‘N’, ‘NE’, ‘E’, ‘SE’, ‘S’, ‘SW’, ‘W’, ‘NW’
conditions	Factor	Survey	Weather conditions during survey: ‘Sunny’, ‘Sunny / Overcast’, ‘Overcast’
cloud	Continuous	Survey	Estimated % of cloud cover at start of survey
temperature	Continuous	Survey	Mean daily temperature (°C) from Jersey Meteorological Section of the Department of the Environment Jersey (linear and quadratic)
transect	Continuous	Site	Transect length (km); used only as an offset on occupancy and abundance
rain	Continuous	Survey	Daily rainfall (mm) from Jersey Meteorological Section of the Department of the Environment Jersey (linear and quadratic)
week	Continuous	Survey	Calendar week with week 1 adjusted to the first week of March (linear and quadratic)

^a^All land-cover covariates were calculated in ArcMap v.10.2.1 (http://arcgis.com).

Within each site, surveys were conducted along a transect encompassing suitable habitats; therefore, the effective sampling area was not equivalent to the whole site. Transects ranged between 0.28 and 19.1 km (mean: 4.66) and were visited up to eight times (mean: 6.95 ± 0.36 s.e.m.; Table 2) per season (March‒October) in 2014 or 2015 by the same surveyor. Transects were surveyed using both visual encounter and ACO methods^31^, with five of the 14 sites surveyed in both years. Repeat surveys of a transect were at least seven days apart to reduce disturbance and behavioural effects upon the study population. To ensure site closure for statistical modelling, a robust survey design is recommended^55^, where repeat site surveys occur over a short time-frame to ensure no change in the population. However, monitoring schemes may conduct surveys over a wide period^22,30^ and so we tested the applicability of occupancy and N-mixture models on data collected in this way. If the closure assumption was violated through random emigration and immigration, the probability of transect occupancy is instead the probability of use, or for abundance, the number of individuals associated with - rather than resident along - a transect^3,17^.

The number of ACOs varied between sites (Table 2). At each site a comparable mixture of roofing felt, corrugated tin and bitumen sheets 0.45–0.5 m^2^ in surface area were used as ACOs to maximise detection in differing thermal conditions^31^. Due to the heterogeneity of the habitats, ACOs were not spaced evenly, but were primarily placed in south-facing habitats away from public disturbance^30^. Covariates thought to influence occupancy, detection or abundance were recorded based upon suspected and known life-history knowledge^20,43^ (Table 3). Data were organised by week to test for effects of survey timing.

Encounters included live individuals, sloughed skins and carcasses. Sloughs and carcasses were removed when found to avoid duplicated records. All captured snakes were photographed for individual identification^12^. The ventral patterns were compared across all captures to calculate a minimum population size based on the maximum number of unique individuals identified. This also provided evidence for validations of site-independence and independence between detections within a survey. This study was approved by the University of Kent School of Anthropology and Conservation ethical review committee. All handling and disturbance was conducted under licence (CR 23), issued under the Conservation of Wildlife Law (Jersey) 2000 by the States of Jersey Department of Environment and in accordance with current guidelines^61^.

### Statistical analysis – detection, occupancy and abundance

We used a static single-season model whereby each site-year combination was treated as a separate site, and transect occupancy or abundance was assumed to be independent for each year. This enabled an effective sample size of 19 ‘site-years’ for determining the influences of covariates upon each parameter with greater precision^17,62^. Within the main text, we refer to ‘site-years’ as sites. Non-independence between site-years could lead to underestimation of model error for occupancy and abundance parameters, so we apply caution in their interpretation. To this single-season dataset we applied a set of hierarchical models, whereby an observation model with the detection/non-detection or count data is conditionally related to a state model describing occupancy or abundance^17^. We assumed that transects had constant occupancy throughout each year and detections between transects were independent^14^. N-mixture models provide estimates of abundance and rely on similar closure assumptions to occupancy models, but instead incorporate count data, assume that no changes in abundance occur across the sampling period and that detections within a survey are independent of each other^9^.

All analyses were conducted in R with packages unmarked^63^ v. 0.11-0 (https://cran.r-project.org/web/packages/unmarked/index.html) and AICcmodavg v. 2.1-0. (https://cran.r-project.org/web/packages/AICcmodavg/index.html). A set of candidate models were developed for each parameter; occupancy, detection or abundance (Table 3; Supplementary Tables S3‒S5). Models were held constant, or allowed to vary by site and observation covariates (detection) or site covariates only (occupancy and abundance). Covariates were incorporated through a logit-link function with only a single covariate for detection or occupancy/abundance for simplicity. Rainfall, temperature and week were incorporated into models as linear and quadratic effects^42^. As transect length and therefore the effective sampling area varied, we incorporated transect length as an offset within each model on occupancy or abundance, assuming they may increase with transect length^17^. Therefore, outputs are the occupancy or abundance per kilometre of transect (density) respectively, unless stated otherwise.

We discarded models that failed to converge, and those remaining were ranked by Akaike weight (*w*_i_) and ΔAICc values as ΔAICc is more appropriate for small sample sizes than ΔAIC^64^. The number of observations was set to 132 (total number of site surveys) for detection and 19 (number of site-years) for occupancy and abundance^17^ (Table 2). Top models were those with ΔAICc ≤ 2^64^, with goodness-of-fit assessed using Pearson’s χ^2^ statistic and 1000 bootstrap simulations^65^. Where overdispersion was indicated ($\hat{c}$ > 1), models were ranked using the quasi-likelihood alternative QAICc^64^ adjusted with the $\hat{c}$ value of the most complex model. For abundance, different mixtures (Poisson, Negative Binomial or Zero-Inflated Poisson) were evaluated via model selection (AICc) and goodness-of-fit tests^42^.

With low detectability and limited sampling occasions, infinite^66^ or biased^6^ estimates of abundance can occur. To avoid this we evaluated varying upper bounds and found 50 to be sufficient. Bayesian approaches were used to estimate the abundance along each transect and total abundance across all transects using the ‘ranef’ and empirical best unbiased predictor (‘BUP’) functions in unmarked^63^. These methods calculate the posterior abundance distribution based on the data and model parameters. Parametric bootstrapping with 1000 simulations was used to identify 95% confidence intervals for total abundance across transects.

### Statistical analysis - survey effort

The minimum number of surveys required to detect a grass snake at an occupied site were calculated for probabilities of 0.80, 0.90 or 0.95^67^. We used detection estimates from our top occupancy model and calculated the minimum number of surveys needed for survey efforts of 0–500 ACOs. These were used to evaluate our own survey efforts and to make recommendations for future monitoring.

Currently, NARRS volunteers survey sites four or more times between March and June. Occupancy data are combined across six-year cycles, where any sites surveyed in multiple years are considered independent. Each six-year cycle is then treated as an independent period and occupancy trends are assessed between the different cycles^30^. Using modified R code from a previous study^38^ (see Supplementary Methods S1), we conducted a one-tailed power analysis to estimate the number of sites required to detect a significant occupancy decline between two independent periods. The probability of Type I (*α*) and Type II (*β*) errors were expected to be under a normal distribution (*z*). The initial (ψ_1_) and resulting (ψ_2_ = ψ_1_ (1 − *R*)) occupancy probabilities were derived from our top model estimates given a transect length of 1.5 km, based on the Jersey NARRS mean^30^. Detection estimates (*p*) were derived from the top detection model.

We varied detection predictions based on the number of ACOs from 0 to 100 at 10 ACO increments, noting that the number of ACOs currently used in Jersey rarely exceeds 10 per site (States of Jersey Department of Environment, pers. comm.). We assessed the number of sites required to detect declines (*R*) of 50%, 30% and 15% using a significance level of α = 0.05 with either four, six or eight survey visits (*K*). We assumed detection and occupancy were constant across seasons and the same number of surveys were made in each period.

### Data availability

The datasets from this study will be made available in the Kent Academic Repository upon the manuscript’s publication.

## Electronic supplementary material

Supplementary Information
