# Supplementary material for: Optimising monitoring efforts for secretive snakes: a comparison of occupancy and N-mixture models for assessment of population status
Source: Sci Rep. 2017 Dec 22;7:18074. doi: 10.1038/s41598-017-18343-5 (PMC5741746; doi:10.1038/s41598-017-18343-5)
Supplement: Supplementary file 1 — Supplementary Information [file 41598_2017_18343_MOESM1_ESM.pdf]

Optimising monitoring efforts for secretive snakes: a comparison of occupancy and N-mixture models for assessment of population status

Robert J. Ward[1,*] and Richard A. Griffiths[1], John W. Wilkinson[2] and Nina Cornish[3]

[1]Durrell Institute of Conservation and Ecology, School of Anthropology and Conservation, University of Kent, Canterbury, Kent, CT2 7NR, UK.

[2]Amphibian and Reptile Conservation, 655a Christchurch Road, Bournemouth, Dorset, BH1 4AP, UK

[3]States of Jersey, Department of the Environment, Howard Davis Farm, La Route de la Trinite, Trinity, Jersey, JE3 5JP, Channel Islands, United Kingdom

*****rjw53@kent.ac.uk

Supplementary Information

Supplementary Table S1. Evaluation of binomial mixture models (Poi = Poisson, NB = negative binomial, ZIP = zero-inflated Poisson) for grass snake abundance with number of observations set to 19. Displayed are model selection criteria (AICc), goodness-of-fit (GoF) statistics from 1000 simulations, and empirical estimates of abundance associated with all transects ($\hat{N}_{total}$) with 95% confidence intervals calculated through 1000 parametric bootstrapping simulations.

Model	Distribution	AICc	Goodness of fit			$\hat{N}_{total}$ (95% CI)
			χ^2	*P*	$\hat{c}$	
p(.), λ(.)	Poi	187.88	250.09	0.01	1.96	39.03 (20–169)
p(.), λ(.)	NB	188.97	257.00	0.02	1.72	51.98 (18–166)
p(.), λ(.)	ZIP	187.87	267.64	0.01	1.86	49.49 (17–156)

Supplementary Table S2. Number of sites to be surveyed in any six-year Jersey NARRS cycle to detect a given occupancy decline (*R*) at a power of 0.8. Results are shown for differing numbers of surveys (*K*), survey effort (numbers of ACOs) and their associated detection (*p*) probability from our top model (Fig. 2). Detection (*p*) and the number of sites needed to detect a decline are shown with 95% confidence intervals. Figures shown in bold indicate numbers of sites ≤ 50 (the number surveyed in the previous six-year Jersey NARRS cycle[1]). Occupancy is held constant based on a transect length of 1.5 km (ψ = 0.54).

ACOs	*p*	*K* = 4			*K* = 6			*K* = 8		
		R = 0.15	*R* = 0.3	*R* = 0.5	*R* = 0.15	*R* = 0.3	*R* = 0.5	*R* = 0.15	*R* = 0.3	*R* = 0.5
0	0.172	3642	843	270[†]	1567	367[†]	118[†]	973	230[†]	75[†]
	(0.084–0.320)	(993–18028)	(235–4148)	(77–1320)	(618–7067)	(149–1630)	(**49**–520)	(524–3783)	(127–876)	(**42**–280)
10*	0.186	3057	709[†]	227[†]	1351	317[†]	103[†]	867	206[†]	67[†]
	(0.095–0.331)	(936–13753)	(222–3166)	(72–1008)	(600–5415)	(145–1251)	(**48**–399)	(516–2929)	(125–680)	(**42**–218)
20	0.200	2601	604[†]	194[†]	1185	279[†]	91[†]	787[†]	187[†]	61[†]
	(0.107–0.342)	(886–10566)	(210–2434)	(69–775)	(583–4189)	(141–969)	(**47**–310)	(509–2299)	(124–535)	(**41**–172)
30	0.215	2219	516[†]	166[†]	1046	247[†]	80[†]	720[†]	172[†]	57[†]
	(0.120–0.353)	(841–8181)	(200–1886)	(65–601)	(569–3278)	(138–760)	(**46**–243)	(503–1833)	(122–428)	(**41**–138)
40	0.231	1899	443[†]	143[†]	932	221[†]	72[†]	666[†]	160[†]	53[†]
	(0.135–0.366)	(795–6282)	(189–1450)	(62–462)	(555–2557)	(134–594)	(**45**–191)	(497–1468)	(121–344)	(**40**–111)
50	0.247	1648	385[†]	124[†]	842	200[†]	66[†]	624[†]	150[†]	**50**[†]
	(0.150–0.379)	(755–4956)	(180–1145)	(59–366)	(542–2057)	(131–479)	(**44**–154)	(493–1217)	(120–286)	(**40**–93)
60	0.264	1436	337[†]	109[†]	768[†]	183[†]	60[†]	590[†]	142[†]	**47**[†]
	(0.166–0.393)	(718–3945)	(172–913)	(56–292)	(531–1679)	(129–393)	(**43**–127)	(488–1029)	(119–243)	(**40**–79)
70	0.283	1250	294[†]	95[†]	704[†]	169[†]	55[†]	561[†]	136[†]	**45**[†]
	(0.183–0.409)	(681–3170)	(163–735)	(54–235)	(520–1393)	(126–327)	(**42**–106)	(484–888)	(118–211)	(**39**–69)
80	0.301	1111	262[†]	85[†]	657[†]	158[†]	52[†]	540[†]	131[†]	**44**[†]
	(0.201–0.425)	(650–2573)	(156–598)	(52–192)	(511–1174)	(124–277)	(**41**–90)	(481–782)	(117–186)	(**39**–61)
90	0.321	988	234[†]	76[†]	616[†]	148[†]	**49**[†]	523[†]	127[†]	**42**[†]
	(0.219–0.444)	(619–2131)	(149–496)	(**49**–160)	(502–1015)	(122–240)	(**41**–78)	(478–705)	(117–169)	(**39**–**56**)
100	0.341	890	211[†]	69[†]	585[†]	141[†]	**47**[†]	510[†]	124[†]	**41**[†]
	(0.237–0.463)	(593–1798)	(143–420)	(**47**–135)	(495–896)	(120–213)	(**40**–69)	(475–649)	(116–156)	(**39**–**51**)

*Number of ACOs used in NARRS surveys 2007–2012 rarely exceeded 10 per site; [†]Mean number of sites ≤ 840; the maximum available in a six-year survey cycle (140 sites*6 years).

Supplementary Table S3. Full set of candidate models with number of observations set to 132 for detection. Models are displayed in descending order and are ranked by their AICc and weight (Wt). K is the number of model parameters and LL is log likelihood. All models have an offset for transect length on the occupancy parameter (not shown).

Models	K	AICc	ΔAICc	AICc Wt	Cum. Wt	LL
p(ACOs), ψ(habitat)	6	124.31	0.00	0.55	0.55	-55.82
p(ACOs), ψ(.)	3	126.47	2.16	0.19	0.74	-60.14
p(ACOs), ψ(aspect)	8	127.46	3.16	0.11	0.86	-55.15
p(ACOs), ψ(ACOs)	4	128.55	4.24	0.07	0.92	-60.12
p(habitat), ψ(.)	5	129.80	5.49	0.04	0.96	-59.66
p(habitat), ψ(ACOs)	6	132.01	7.70	0.01	0.97	-59.67
p(habitat), ψ(habitat)	8	133.07	8.76	0.01	0.98	-57.95
p(aspect), ψ(.)	7	133.38	9.07	0.01	0.98	-59.24
p(aspect), ψ(habitat)	10	134.77	10.46	0.00	0.99	-56.47
p(temperature)+(temperature^2), ψ(habitat)	7	135.74	11.44	0.00	0.99	-60.42
p(.), ψ(habitat)	5	135.96	11.65	0.00	0.99	-62.74
p(temperature)+(temperature^2), ψ(.)	4	136.38	12.08	0.00	0.99	-64.03
p(.), ψ(.)	2	136.54	12.23	0.00	0.99	-66.22
p(cloud), ψ(habitat)	6	136.80	12.49	0.00	0.99	-62.06
p(cloud), ψ(.)	3	137.09	12.78	0.00	0.99	-65.45
p(.), ψ(aspect)	7	137.64	13.34	0.00	0.99	-61.37
p(temperature)+(temperature^2), ψ(aspect)	9	137.73	13.43	0.00	1.00	-59.13
p(aspect), ψ(ACOs)	8	138.26	13.95	0.00	1.00	-60.54
p(temperature)+(temperature^2), ψ(ACOs)	5	138.31	14.01	0.00	1.00	-63.92
p(cloud), ψ(aspect)	8	138.37	14.07	0.00	1.00	-60.60
p(.), ψ(ACOs)	3	138.40	14.10	0.00	1.00	-66.11
p(cloud), ψ(ACOs)	4	139.01	14.70	0.00	1.00	-65.35
p(week)+(week^2), ψ(habitat)	7	139.04	14.73	0.00	1.00	-62.07
p(rain)+(rain^2), ψ(habitat)	7	139.10	14.79	0.00	1.00	-62.10
p(rain)+(rain^2), ψ(.)	4	139.32	15.01	0.00	1.00	-65.50
p(week)+(week^2), ψ(.)	4	139.89	15.58	0.00	1.00	-65.79
p(condition), ψ(habitat)	7	139.99	15.69	0.00	1.00	-62.54
p(rain)+(rain^2), ψ(aspect)	9	140.16	15.86	0.00	1.00	-60.34
p(condition), ψ(.)	4	140.44	16.13	0.00	1.00	-66.06
p(habitat), ψ(aspect)	10	140.68	16.37	0.00	1.00	-59.43
p(week)+(week^2), ψ(aspect)	9	140.73	16.42	0.00	1.00	-60.63
p(rain)+(rain^2), ψ(ACOs)	5	141.21	16.90	0.00	1.00	-65.37
p(week)+(week^2), ψ(ACOs)	5	141.73	17.42	0.00	1.00	-65.63
p(condition), ψ(aspect)	9	141.81	17.50	0.00	1.00	-61.17
p(condition), ψ(ACOs)	5	142.37	18.06	0.00	1.00	-65.95

Supplementary Table S4. Full set of candidate models with number of observations set to 19 for occupancy. Models are displayed in descending order and are ranked by their AICc and weight (Wt). K is the number of model parameters and LL is log likelihood. All models have an offset for transect length on the occupancy parameter (not shown).

Models	K	AICc	ΔAICc	AICc Wt	Cum. Wt	LL
p(ACOs), ψ(.)	3	127.88	0.00	0.65	0.65	-60.14
p(ACOs), ψ(habitat)	6	130.63	2.75	0.16	0.81	-55.82
p(ACOs), ψ(ACOs)	4	131.09	3.21	0.13	0.95	-60.12
p(habitat), ψ(.)	5	133.94	6.06	0.03	0.98	-59.66
p(.), ψ(.)	2	137.20	9.32	0.01	0.98	-66.22
p(habitat), ψ(ACOs)	6	138.34	10.46	0.00	0.99	-59.67
p(cloud), ψ(.)	3	138.50	10.62	0.00	0.99	-65.45
p(temperature)+(temperature^2), ψ(.)	4	138.93	11.04	0.00	0.99	-64.03
p(.), ψ(ACOs)	3	139.82	11.93	0.00	0.99	-66.11
p(.), ψ(habitat)	5	140.10	12.22	0.00	1.00	-62.74
p(ACOs), ψ(aspect)	8	140.69	12.81	0.00	1.00	-55.15
p(cloud), ψ(ACOs)	4	141.55	13.67	0.00	1.00	-65.35
p(rain)+(rain^2), ψ(.)	4	141.86	13.98	0.00	1.00	-65.50
p(week)+(week^2), ψ(.)	4	142.43	14.55	0.00	1.00	-65.79
p(temperature)+(temperature^2), ψ(ACOs)	5	142.45	14.57	0.00	1.00	-63.92
p(aspect), ψ(.)	7	142.66	14.78	0.00	1.00	-59.24
p(condition), ψ(.)	4	142.98	15.10	0.00	1.00	-66.06
p(cloud), ψ(habitat)	6	143.13	15.25	0.00	1.00	-62.06
p(temperature)+(temperature^2), ψ(habitat)	7	145.02	17.14	0.00	1.00	-60.42
p(rain)+(rain^2), ψ(ACOs)	5	145.35	17.47	0.00	1.00	-65.37
p(week)+(week^2), ψ(ACOs)	5	145.87	17.99	0.00	1.00	-65.63
p(habitat), ψ(habitat)	8	146.30	18.42	0.00	1.00	-57.95
p(condition), ψ(ACOs)	5	146.51	18.63	0.00	1.00	-65.95
p(.), ψ(aspect)	7	146.92	19.04	0.00	1.00	-61.37
p(week)+(week^2), ψ(habitat)	7	148.32	20.44	0.00	1.00	-62.07
p(rain)+(rain^2), ψ(habitat)	7	148.37	20.49	0.00	1.00	-62.10
p(condition), ψ(habitat)	7	149.27	21.39	0.00	1.00	-62.54
p(aspect), ψ(ACOs)	8	151.48	23.60	0.00	1.00	-60.54
p(cloud), ψ(aspect)	8	151.60	23.72	0.00	1.00	-60.60
p(temperature)+(temperature^2), ψ(aspect)	9	156.26	28.38	0.00	1.00	-59.13
p(rain)+(rain^2), ψ(aspect)	9	158.69	30.81	0.00	1.00	-60.34
p(week)+(week^2), ψ(aspect)	9	159.25	31.37	0.00	1.00	-60.63
p(condition), ψ(aspect)	9	160.33	32.45	0.00	1.00	-61.17
p(aspect), ψ(habitat)	10	160.45	32.57	0.00	1.00	-56.47
p(habitat), ψ(aspect)	10	166.36	38.48	0.00	1.00	-59.43

Supplementary Table S5. Full set of candidate models with number of observations set to 19 for abundance and the Poisson mixture. Models are displayed in descending order and are ranked by their QAICc and weight (Wt) following a correction with $\hat{c}$ = 1.16. K is the number of model parameters and LL is log likelihood. All models have an offset for transect length on the abundance parameter (not shown).

Models	K	QAICc	ΔQAICc	QAICc Wt	Cum. Wt	LL
p(.), λ(.)	3	166.11	0.00	0.42	0.42	-79.26
p(.), λ(ACOs)	4	168.71	2.60	0.12	0.54	-78.93
p(ACOs), λ(.)	4	169.21	3.10	0.09	0.63	-79.18
p(cloud), λ(.)	4	169.29	3.18	0.09	0.71	-79.22
p(temperature)+(temperature^2), λ(.)	5	170.25	4.14	0.05	0.77	-77.82
p(condition), λ(.)	5	170.26	4.15	0.05	0.82	-77.82
p(rain)+(rain^2), λ(.)	5	170.45	4.34	0.05	0.87	-77.92
p(ACOs), λ(ACOs)	5	171.10	4.98	0.04	0.90	-78.24
p(cloud), λ(ACOs)	5	172.38	6.26	0.02	0.92	-78.88
p(.), λ(habitat)	6	172.41	6.30	0.02	0.94	-76.71
p(week)+(week^2), λ(.)	5	172.50	6.38	0.02	0.96	-78.94
p(habitat), λ(.)	6	173.83	7.71	0.01	0.97	-77.41
p(condition), λ(ACOs)	6	173.87	7.75	0.01	0.98	-77.43
p(temperature)+(temperature^2), λ(ACOs)	6	173.98	7.87	0.01	0.98	-77.49
p(rain)+(rain^2), λ(ACOs)	6	174.10	7.99	0.01	0.99	-77.55
p(week)+(week^2), λ(ACOs)	6	176.23	10.11	0.00	0.99	-78.61
p(ACOs), λ(habitat)	7	176.44	10.33	0.00	1.00	-76.13
p(cloud), λ(habitat)	7	177.53	11.42	0.00	1.00	-76.68
p(habitat), λ(ACOs)	7	178.95	12.83	0.00	1.00	-77.38
p(condition), λ(habitat)	8	180.60	14.49	0.00	1.00	-75.10
p(temperature)+ (temperature^2), λ(habitat)	8	181.22	15.10	0.00	1.00	-75.41
p(aspect), λ(.)	8	181.25	15.13	0.00	1.00	-75.42
p(rain)+(rain^2), λ(habitat)	8	181.41	15.29	0.00	1.00	-75.50
p(.), λ(aspect)	8	181.90	15.79	0.00	1.00	-75.75
p(week)+(week^2), λ(habitat)	8	183.25	17.13	0.00	1.00	-76.42
p(aspect), λ(ACOs)	9	186.99	20.88	0.00	1.00	-74.50
p(habitat), λ(habitat)	9	188.53	22.41	0.00	1.00	-75.26
p(ACOs), λ(aspect)	9	189.07	22.95	0.00	1.00	-75.53
p(cloud), λ(aspect)	9	189.40	23.29	0.00	1.00	-75.70
p(rain)+(rain^2), λ(aspect)	10	196.01	29.90	0.00	1.00	-74.25
p(condition), λ(aspect)	10	196.34	30.23	0.00	1.00	-74.42
p(temperature)+(temperature^2), λ(aspect)	10	196.80	30.69	0.00	1.00	-74.65
p(week)+(week^2), λ(aspect)	10	197.87	31.76	0.00	1.00	-75.19
p(aspect), λ(habitat)	11	200.51	34.40	0.00	1.00	-70.40
p(habitat), λ(aspect)	11	203.59	37.48	0.00	1.00	-71.94

Supplementary Methods S1. R code for conducting a one-tailed power analysis of occupancy data (modified from a previous study[2]).

```
# Function 'calcSFormula' for to calculate the number of sites needed to detect an occupancy decline at a given power
    calcSFormula <- function(K1,K2,p1,p2,psi1,R,alpha,pow)
    {
     psi2 <-psi1*(1-R)
     pp1 <- 1-(1-p1)^K1
     pp2 <- 1-(1-p2)^K2
     F1 <- (1-pp1)/(pp1-K1*p1*(1-p1)^(K1-1))
     F2 <- (1-pp2)/(pp2-K2*p2*(1-p2)^(K2-1))
     f1 <- psi1*(1-psi1+F1)
     f2 <- psi2*(1-psi2+F2)
     S <- (f1+f2)*((qnorm(1-alpha)+qnorm(pow))/(psi1-psi2))^2
     return(ceiling(S))
}
# Set the parameters (values given are examples)
     K<- 4 # number of replicates
     p <- 0.186 # detection probability
     psi1 <- 0.50 # initial occupancy probability
     R <- 0.5 # proportional change in occupancy. Positive value for a decrease, negative value for an increase.
     alpha <- 0.05 # significance level
     pow <- 0.8 # target power level

# Perform the calculation
(SS <- calcSFormula(K1 = K, K2 = K, p1 = p, p2 = p, psi1, R, alpha, pow))
```

References

1. Wilkinson, J. W., French, G. C. & Starnes, T. *Jersey NARRS report 2007 - 2012. Results of the first full NARRS cycle in Jersey: setting the baseline. Unpublished report to the States of Jersey Environment Department.* (Amphibian and Reptile Conservation Trust, 2013). Available at: https://www.gov.je/SiteCollectionDocuments/Government%20and%20administration/R%20-%20Jersey%20National%20Amphibian%20and%20Reptile%20Recording%20Scheme%20Report%202007-12%20DM%2021052015.pdf. (Date of access: 13/03/2014).

2. Guillera-Arroita, G. & Lahoz-Monfort, J. J. Designing studies to detect differences in species occupancy: power analysis under imperfect detection. *Methods Ecol. Evol.* **3**, 860-869 (2012).
